# The Men's Safer Sex (MenSS) trial: protocol for a pilot randomised controlled trial of an interactive digital intervention to increase condom use in men

**DOI:** 10.1136/bmjopen-2014-007552

**Published:** 2015-02-16

**Authors:** Julia V Bailey, Rosie Webster, Rachael Hunter, Nick Freemantle, Greta Rait, Susan Michie, Claudia Estcourt, Jane Anderson, Makeda Gerressu, Judith Stephenson, Chee Siang Ang, Graham Hart, Sacha Dhanjal, Elizabeth Murray

**Affiliations:** 1 eHealth Unit, Research Department of Primary Care and Population Health, University College London, London, UK; 2 PRIMENT Clinical Trials Unit, Research Department of Primary Care and Population Health, University College London, London, UK; 3 Research Department of Clinical, Educational, and Health Psychology, University College London, London, UK; 4 BICMS, Barts and The London School of Medicine & Dentistry, Barts Sexual Health Centre, St Bartholomew's Hospital, London, UK; 5 Homerton Sexual Health Services, Homerton University Hospital, London, UK; 6 Centre for Sexual Health and HIV Research, University College London, London, UK; 7 School of Engineering and Digital Arts, University of Kent, Canterbury, UK; 8 Department of Infection & Population Health, University College London, London, UK

**Keywords:** sexual health, randomized controlled trial, online trial, interactive digital intervention, behavior change, complex intervention

## Abstract

**Introduction:**

Sexually transmitted infections (STI) are a major public health problem. Condoms provide effective protection but there are many barriers to use. Face-to-face health promotion interventions are resource-intensive and show mixed results. Interactive digital interventions may provide a suitable alternative, allowing private access to personally tailored behaviour change support. We have developed an interactive digital intervention (the Men's Safer Sex (MenSS) website) which aims to increase condom use in men. We describe the protocol for a pilot trial to assess the feasibility of a full-scale randomised controlled trial of the MenSS website in addition to usual sexual health clinical care.

**Methods and analysis:**

Participants: Men aged 16 or over who report female sexual partners and recent unprotected sex or suspected acute STI. Participants (N=166) will be enrolled using a tablet computer in clinic waiting rooms. All trial procedures will be online, that is, eligibility checks; study consent; trial registration; automated random allocation; and data submission. At baseline and at 3, 6 and 12 months, an online questionnaire will assess condom use, self-reported STI diagnoses, and mediators of condom use (eg, knowledge, intention). Reminders will be by email and mobile phone. The primary outcome is condom use, measured at 3 months. STI rates will be recorded from sexual health clinic medical records at 12 months. The feasibility of a cost-effectiveness analysis will be assessed, to calculate incremental cost per STI prevented (Chlamydia or Gonorrhoea), from the NHS perspective.

**Ethics and dissemination:**

Ethical approval: City and East NHS Research Ethics Committee (reference number 13 LO 1801). Findings will be made available through publication in peer-reviewed journals, and to participants and members of the public via Twitter and from the University College London eHealth Unit website. Raw data will be made available on request.

**Trial registration number:**

Current Controlled Trials. ISRCTN18649610. Registered 15 October 2013 http://www.controlled-trials.com/ISRCTN18649610.

## Introduction

### Men's sexual health

Sexually transmitted infections (STI) are a major public health problem, with high social and economic costs.^1^ ^2^ Condoms are effective for prevention of STI; however, there are many barriers to successful use, for example, decrease in sensation, interruption of sex, incorrect size or fit, use of alcohol/recreational drugs, anxiety affecting sexual performance, and stigma associated with carrying condoms.^3^ ^4^ The prevention of pregnancy is often a stronger motivation for condom use than prevention of STI.^5^ Condoms may be perceived as a barrier to intimacy and trust,^4^ and use is often lower in established relationships.^6^

Since it is men who primarily experience many of the disadvantages of using male condoms (eg, reduced pleasure), and have the power to influence condom use for penetrative sex (since they wear condoms), prevention efforts are needed to target the obstacles to condom use that men face.^3^ While there are a variety of health promotion interventions aimed at improving sexual health for men who have sex with men (MSM), there are fewer interventions specifically for adult men who have sex with women (MSW),^7^ ^8^ despite the fact that MSW report much less consistent condom use than MSM.^9^ Men are less likely than women to visit health professionals and generally have shorter clinic appointments,^10^ ^11^ so they may be less likely to be offered health promotional advice or risk reduction counselling in the context of routine appointments. Men may be reluctant to discuss their sexual health with health professionals, partners or friends.^12^ An online intervention therefore offers an alternative avenue to reach men.^13^

### Sexual health interventions

Guidance from the National Institute for Health and Care Excellence recommends that people at high risk of STI are offered one-to-one structured discussions to address risk-taking,^1^ and this is increasingly being offered as part of routine care in genitourinary medicine and other healthcare settings. While interventions such as motivational interviewing can impact on sexual behaviour,^1^ in practice it is resource-intensive to train and support staff, and difficult to find time for structured discussions in busy clinical services. A potential alternative to such interventions is the use of interactive digital interventions (IDI).

We define IDI as ‘Computer-based programmes that provide information and one or more of decision support, behaviour change support, or emotional support for health issues’.^13^ IDI require contributions from users to produce personally relevant tailored material and feedback. IDI are highly suitable for sexual health promotion because access can be private, anonymous and self-paced,^14^ which may be particularly important for men who may be reluctant to disclose a lack of knowledge or skill. Interventions can be targeted for specific groups (eg, by age, gender or sexuality), and content can be tailored for individuals.^15^ IDI can be expensive to develop but offer the advantages of intervention fidelity^16^ and the potential to reach large audiences at relatively low dissemination costs.

IDI can improve sexual behaviour (including condom use),^13^ ^17^ as well as increasing knowledge, self-efficacy and safer sex intention.^13^ ^18^ More evidence is needed to establish effects on biological outcomes (STI) and cost-effectiveness. The Men's Safer Sex (MenSS) website is an IDI which aims to increase condom use and reduce STI in men attending sexual health clinics. The present pilot trial aims to determine the optimum parameters of a full-scale randomised controlled trial (RCT) to assess the efficacy of the MenSS website intervention.

## Objectives

To establish the feasibility and optimal design of a full-scale RCT to test the effect on condom use and STI acquisition of the MenSS intervention website for men attending sexual health clinics:

- Conduct a pilot trial to optimise the parameters for a phase III RCT of usual clinical care plus the IDI compared to usual clinical care only, using the primary outcome of self-reported condom use at 3-month follow-up.
- Optimise the data collection and analysis procedures for a health economic analysis for a future Phase III RCT.

### Methods and analysis

The pilot trial will be a phase II proof of concept RCT to evaluate the effect of the MenSS IDI on increasing condom use in addition to usual clinic care for men in sexual health clinics.

### Setting

Participants will be recruited from three sexual health clinics: The Homerton Hospital Department of Sexual Health, St Bartholomew's Sexual Health Centre, and City of Coventry Health Centre Integrated Sexual Health Services Department. These clinics serve a diverse range of patients in terms of age, socioeconomic status and ethnicity.

### The intervention

The MenSS website content and design was developed based on evidence from the sexual health research literature and theories of behaviour change, qualitative interviews with men in sexual health clinics, and discussions with clinical and academic experts in sexual health and digital technologies.^19^ The development process was iterative, with a high level of user involvement. The intervention is designed to be delivered initially in clinic, to make use of the time when patients are waiting to be seen, but also providing (and encouraging) online access after patients have left the clinic.

The MenSS intervention consists of an interactive website. While in clinic, users will be presented with a tailored package of website content which addresses individual men's barriers to condom use. The site targets a number of influences on effective condom use, such as:

- Condom knowledge (eg, about sizes and types of condoms);
- Condom use skills;
- Difficulties in negotiating condom use;
- Inaccurate beliefs about STI risk;
- Social influences, such as perceived/expected partner response;
- Sexual pleasure;
- Being caught in the ‘heat of the moment’;
- Alcohol and drug use.

While in clinic, users will be asked to select their own personal barriers to condom use. This task will produce a tailored package of information, offering solutions or counter-arguments to barriers (including interactive activities and quizzes, videos and case vignettes), which will be presented prominently on the homepage. Some content is presented to all users (eg, training in condom use skills), and all content will be available via website navigation tabs. Participants will be led through their tailored content package sequentially, and will be asked to set goals to change their behaviour. If wanted, participants will receive emails to assess achievement of goals, and to encourage them to visit the intervention website again.

### Procedure

**Figure 1:**
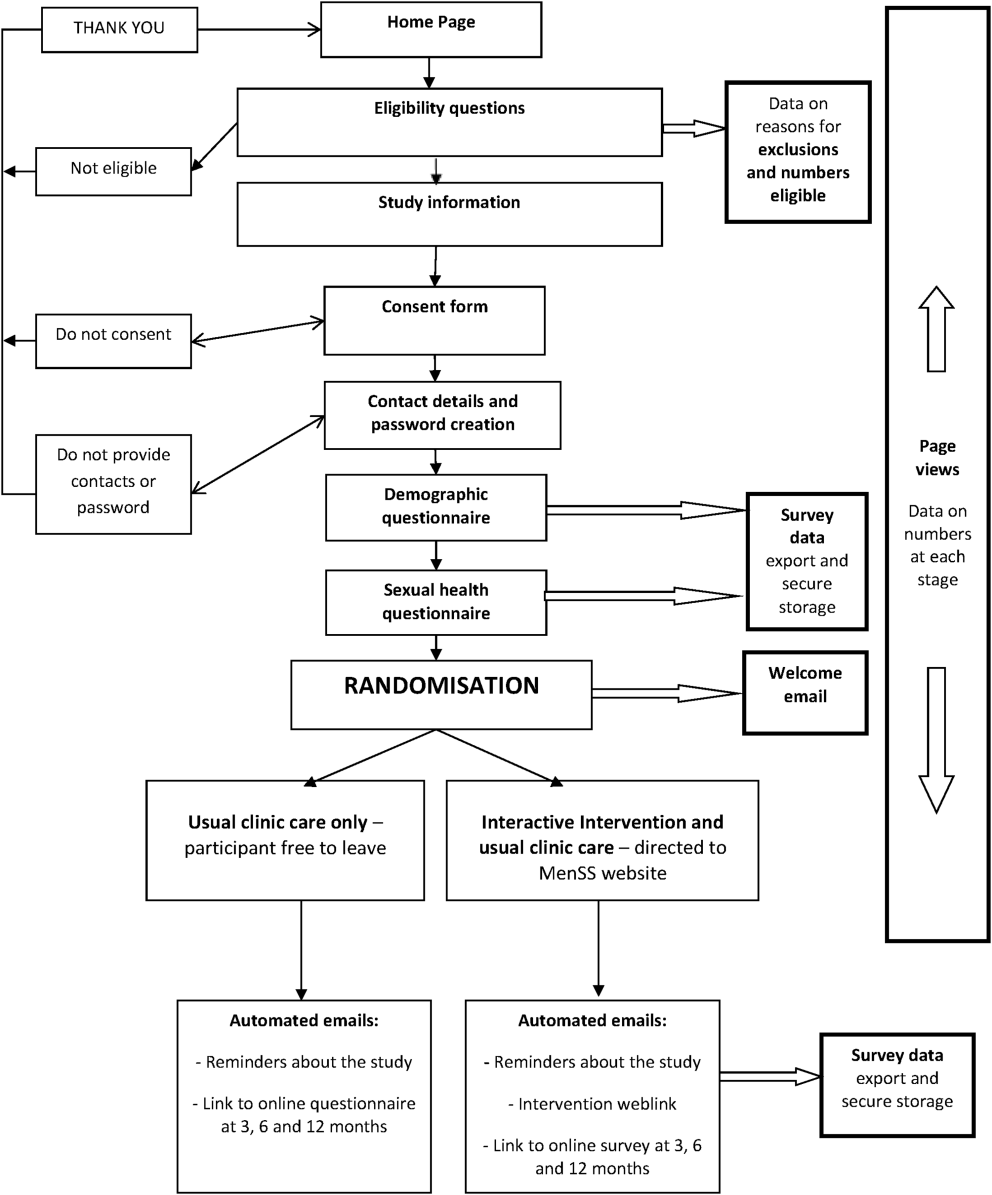
Men's Safer Sex trial software framework: online study information, consent, registration, data collection and randomisation.

Participant recruitment is designed to be self-directed, using a touch screen tablet computer, which will be available in the clinic waiting room. Participants will be directed to the tablet computer via brief information leaflets and posters in the waiting room, or by clinic staff. The trial software is set up to allow participants to be led through the steps of screening, consent, automatic randomisation, data collection and intervention viewing, without assistance from clinic staff (figure 1). A member of the study team or the sexual health clinic research staff will be present if needed to provide technical assistance and to answer questions about the study.

#### Online eligibility, consent, registration, randomisation, data collection

The tablet computer in the clinic waiting room will present information about the trial and an invitation to participate. Potential participants will then be asked to complete a screening questionnaire to determine their eligibility for the trial. Those who are ineligible at this point will be informed of this, and thanked for their time. Those who are eligible will receive detailed information regarding the study. Participants will be asked to give informed consent by agreeing to a number of statements (see online supplementary appendix 1: online participant information and consent). They will then be asked to give their contact details (email address and telephone number), in order to contact them for follow-up assessments, to send them reminders to view the website, to remind them that they are participating in the trial, and to send an electronic shopping voucher to recompense participants for their time. Participants will also be asked to create a password. The password will give the intervention group access to the intervention website, which will also enable collection of website usage data. It will also be needed to access the follow-up questionnaires. Demographic and baseline sexual health data will then be collected. At this point, the software program will use a computer algorithm to randomly allocate participants to either the intervention or control conditions. This allocation will be unalterable.

#### Follow-up data collection

At 3, 6 and 12 months after their initial clinic visit, participants will receive an automated email asking them to complete a follow-up questionnaire (see Outcome assessment section), by clicking on a weblink to an online questionnaire. If they do not complete the questionnaire, they will receive three further email prompts, at 1-week intervals, as well as two text messages to their mobile phone (again including the weblink) alongside the latter two emails. If participants still do not respond, the researcher will telephone participants a week after the final email to remind them to fill in the study questionnaire. Information about STI diagnoses will be collected at all time points via self-report, and at 12 months by recording diagnoses or suspected diagnoses over the past year recorded in the clinical notes at the sexual health clinics participants are recruited from.

### Inclusion and exclusion criteria

#### Inclusion criteria

Men aged 16 years and above (with no upper age limit); able to read English; with access to the internet; and at high risk of future STI (ie, two or more partners in the past year (male or female) and some non-condom use in the past 3 months; or symptoms of acute STI; or seeking treatment for an STI); and for whom at least half of their sexual partners are female.

#### Exclusion criteria

HIV-positive men and men with hepatitis B or C will be excluded, since patients with these diagnoses are likely to receive risk reduction advice in the course of routine clinical care. We will exclude men who have had sexual experience only ever with males, more often with males but at least once with a female, or no sexual experience at all.^20^

### Randomisation

Once participants have been checked for eligibility, given informed consent, and submitted baseline data, they will be allocated by the computer algorithm randomisation system to either the intervention or control group. The participant will be informed with an automated message on the tablet computer, and this allocation will be unalterable.

### Allocation concealment

Allocation will be undertaken using a concealed automated computer-based algorithm, which will be immediate.

Participants allocated to the control condition will be notified that they have not been selected to view the intervention and told that they will be contacted again in 3, 6 and 12 months to gather follow-up data. Those allocated to the intervention condition will be directed to the intervention website where they will be presented with a tailored package of health promotion/behaviour change content. Website usage (page views) will be automatically recorded.

### Outcome assessment

#### Development of the online measurement instrument

We adapted the Sexunzipped online sexual health questionnaire^21^ to measure outcomes and mediators of condom use. We selected items for inclusion based on a literature search for established measures, and consultation with experts. We conducted interviews with men in sexual health clinics (N=11) to gain feedback on successive versions of the outcome questionnaire. Interviews with men checked their understanding of questions, the clarity of questions, and content suitability for the selected measures of behavioural outcomes and mediators of those outcomes. On the basis of feedback, we modified the structure and content of the outcome questionnaire.

Mediators of behaviour change (eg, beliefs about pleasure, motivation, knowledge, self-efficacy), behavioural outcomes (including condom use, STI testing, communication with partner/s) and cumulative STI incidence (self-reported and from clinical notes) will be measured. Service use and quality of life will be measured for the cost-effectiveness analysis. The primary outcome will be self-reported condom use at 3-month follow-up.

#### Measures

In the light of evidence that measurement alone may prompt behaviour change,^22^ we will measure a limited number of outcomes (condom use and the main mediators, and self-reported STI diagnoses) at baseline; we will also assess a full range of outcomes at 3, 6 and 12 months (table 1). All assessments have a recall period of the previous 3 months, which is the time between follow-up assessments.

**Table 1:** Variables assessed at each time point

Baseline measures	3-month, 6-month and 12-month follow-up assessments
Demographic details (age, occupation, ethnicity)	
**Sexual health outcomes**	
Sexual partners	Sexual partners
Condom use—episodes and partners	Condom use—episodes and partners
Self-reported STI diagnoses	Self-reported STI diagnoses
Contraception use and pregnancy	Contraception use and pregnancy
Health-related quality of life	Health-related quality of life
	Service use
**Mediators of condom use**	
Motivation to use condoms	Motivation to use condoms
Intentions to use condoms	Intentions to use condoms
Beliefs about pleasure	Beliefs about pleasure
Non-condom use due to intoxication	Non-condom use due to intoxication
	Evaluation of condom use
	Communication
	Identity
	Self-efficacy
	Condom problems
	Knowledge

#### Outcome measures

The online outcome measurement instrument is detailed in full in online supplementary appendix 2.

##### Demographics

Questions at baseline will collect demographic information including age, employment status and ethnicity.

##### Condom use

The objective of the study is to promote condom use with female partners, so the primary outcome is the number of episodes of unprotected vaginal sex (without a condom) over the previous 3 months, assessed at the 3-month follow-up. We expect the majority of change in behaviour to occur shortly after recruitment, as we expect users will be most likely to engage with the intervention during the clinic visit.

##### Sexual partners

Participants will be asked to report the number and type of sexual partners over the past 3 months (both female and male). We will also assess the number of partners participants have had unprotected sex with over the previous 3 months: female (vaginal and anal sex) and male (anal sex).

##### Contraception use and pregnancy

Participants will be asked to indicate which types of contraception (if any) they are using with current partners. Participants will be asked whether a female partner has been pregnant in the past 3 months, and the outcome of that pregnancy (if known).

##### STI diagnoses

Participants will be asked to report STI diagnoses over the past 3 months at every follow-up point. We will also assess whether participants have received treatment due to a partner being diagnosed with an STI. In order to assess laboratory diagnoses, all STI diagnoses recorded in sexual health clinic records (in the participating sites) over the study period will be noted at 12 months.

##### Health-related quality of life

Health-related quality of life (HRQoL) will be assessed using the EQ-5D,^23^ ^24^ which is a 5-item, 3-level questionnaire covering self-care, usual activity, anxiety and depression, and pain and mobility. We will also use a newly developed sexual health Quality of Life Scale,^25^ and compare its performance with the EQ-5D, to assess its suitability for outcome assessment in a sexual health context.

##### Service use

Use of a variety of sexual health services over the period of the study will be assessed (eg, sexual health clinics, general practice, outreach services).

#### Measuring mediators of condom use

While it is important to assess changes in behaviour, it is also important to assess the mediators of behaviour change. This provides information about the mechanisms by which behaviour might have changed. The mediators measured were identified following consultation with experts, a review of the literature, interviews with the target population, and using the theoretical frameworks of the COM-B model^26^ and the PRIME theory of motivation.^27^

##### Condom use errors and problems

To ensure that condom use is ‘correct’, and to assess any impact on condom use skills, we will assess condom use problems at all time points, using a measure defined by Crosby *et al*,^28^ which assesses the occurrence of 15 condom errors and problems within the past 3 months. The scale was adapted in the light of qualitative fieldwork, to improve relevance and clarity.

##### Knowledge

Knowledge (of risk of STIs and condom sizes) will be assessed using an 11-item measure, devised based on gaps in men's knowledge identified in the literature and in interviews with the target population. A number of ‘true or false’ statements regarding misconceptions about condoms and risk will be given (eg, ‘You would know if you had an STI, without needing a test’; ‘Standard sized condoms are suitable for all men’).

##### Communication with partners

To assess communication with partners over a 3-month period, we adapted the six-item Partner Communication Scale.^29^ The scale was adapted in the light of qualitative fieldwork, and the recall period was modified from 6 to 3 months, as follow-up assessments will be 3 months apart.

##### Identity

To assess potential links between identity (self-perception) and condom use,^27^ we created a seven-item scale, derived from issues relating to condom use identity that had been identified during the fieldwork.

##### Beliefs about pleasure

Beliefs about pleasure will be assessed using an eight-item scale, adapted from the ‘Effect of sexual experience’ subscale of the Condom Perceived Barriers Scale.^30^ The scale was adapted in the light of qualitative fieldwork, to improve relevance and clarity.

##### Self-efficacy

Self-efficacy will be assessed using a 14-item measure, adapted from the widely validated Brafford and Beck Scale.^31^ The scale was adapted in the light of qualitative fieldwork, to improve relevance and clarity.

##### Motivation, intention and evaluation of condom use

Motivation (want) and intention to use condoms, and evaluation of condom use will be assessed using single-item measures (Robert West, 12 September 2013, personal communication).

##### Alcohol and drug use

Alcohol and drug use were found in our fieldwork to be important factors in non-condom use. We therefore included a single item assessing the number of times in the past 3 months that participants had unprotected sex when intoxicated.

#### Engagement with the intervention (patterns of website use)

We will record website usage in order to assess engagement with the intervention (and whether this appears to be related to outcomes). The software used will record the number of times each user visits the site, the pages visited and the time spent on each topic section.

#### Adverse effects

We will record any adverse impacts on sexual health outcomes at 3, 6 and 12 months. Participants will be asked to report whether they have experienced any adverse impacts as a result of the study, recording this in a free text box on each of the follow-up questionnaires. Adverse impacts may also be identified when the research team liaise directly with participants (eg, participant emails or follow-up telephone calls to non-responders to questionnaires).

#### Intervention development costs and trial feasibility indicators

Intervention development costs and recruitment and retention rates will be reported.

### Methods to protect against sources of bias

Participants will use the tablet computer without assistance, providing baseline data that will be submitted directly online. A study researcher will be available in the clinic and via telephone, solely to clarify research procedures and assist with technical problems. Baseline data will be collected prior to randomisation. Once eligibility for the study is established and baseline data are collected, allocation to the intervention or control group will be automatically randomly assigned by computer algorithm, and this will not be changeable by participants or researchers. Subsequent outcome data will be collected online using an emailed link to the online outcome questionnaire. Data will be exported and analysed using ID numbers only. Participants will be aware of their allocation to the intervention or control group, but the automated data collection procedures protect from researcher bias during data collection.

### Maximising retention

We will follow up participants by email, texts and by telephone:^32^ ^33^

1. Automated emails, with three further follow-up emails at weekly intervals;
2. Two text messages—at the same time as the last two emails;
3. Contact via telephone a week after the final email.

We will offer participants a £10 online shopping voucher for filling in the online questionnaires at 3 and 6 months, with a £20 voucher for the final 12-month follow-up questionnaire. Vouchers will be sent by email.

## Statistical analysis plan

### Sample size and power calculations

This is a pilot RCT with a primary aim of assessing the success of recruitment and retention, engagement with the intervention, and the acceptability of trial procedures to participants and clinic staff. We will also calculate the effect sizes of key outcomes including condom use over the past 3 months, to inform power calculations for a future phase III RCT.

The study is powered to allow estimates of the effect of the intervention on episodes of unprotected vaginal sex over the past 3 months. A sample size of 166 participants (83 intervention, 83 comparator, randomised 1:1 between experimental and control conditions) is adequate to detect a reduction of 1.35 episodes of unprotected sex with a conventional two-sided α of 0.05 and 90% power (1−β). Allowing for potential loss to follow-up at 3 months, a sample size of 122 participants (61 intervention, 61 comparator) is adequate to find a reduction of 1.35 episodes of unprotected sex with a conventional two-sided α of 0.05 and 80% power (1−β). In addition, this sample size is also sufficient to detect a 1.65 difference in safer sex intention, and a one-point difference in self-efficacy on Likert scales, with a conventional two-sided α of 0.05 and 90% power (1−β).

### Data analysis

#### Analysis of outcomes

Analysis of sexual health outcomes will be based on all participants according to their initial experimental allocation (on an intention-to-treat analysis). Analysis for the primary outcome will use a generalised mixed model, with log link and Poisson/mixed error. The response variable will be the number of episodes of unprotected vaginal sex for each subject. Explanatory variables will be the baseline (log_e_(x)) number of episodes of unprotected vaginal sex and the experimental condition. The analysis will include a generalised (random effects) over-dispersion parameter. Comparisons of sexual health between intervention and control groups will include the baseline value of each outcome as subject level explanatory variables, so that analysis is of the difference in end point conditional on the within-subject baseline measure. For sexual health outcomes measured only at follow-up, we will compare effect sizes between intervention and control groups alone using all available data and describe loss to follow-up for each treatment condition. For other outcomes, analyses will be based on generalised linear models with appropriate link functions and error structures. Statistical analyses will be described a priori in a Statistical Analysis Plan, and the principal analyses will be implemented independently by two statisticians.

#### Mediation analyses

We will conduct a prognostic model in order to determine whether change in any of the mediating variables (eg, beliefs about pleasure, self-efficacy) is associated with any intervention effects. This will help to identify which elements of the website seem to be most influential.

### Cost-effectiveness analysis

The principal aim of the economic data collection will be to determine the feasibility and validity of collecting cost and outcome data for a cost-effectiveness analysis within a phase III trial. We will conduct an initial cost-effectiveness analysis (CEA) of incremental cost per gain in outcome, looking at the cost per STI prevented (*Chlamydia* or *Gonorrhoea*), comparing intervention participants with controls from the NHS perspective. This will include one-way, two-way and parametric sensitivity tests.

The aim of the analysis will primarily be to evaluate whether information collected is fit for the purpose, and to inform information collection in a future trial. We will examine the feasibility of collecting cost data for intervention and control participants including costs associated with STI tests and treatments, and contract tracing, testing and treatment. Trial subjects may access sexual health services from a range of providers, so information from sexual health clinic notes alone may prove unreliable. We will therefore ask participants about sexual health-related health service contacts over the past 12 months as part of the self-reported outcomes. Information collected from sexual health clinic notes will be used to assess the reliability of the self-reported information collected. Personal Social Services Research Unit (PSSRU) reference costs,^34^ British National Formulary^35^ and other national sources of costing information will be used to calculate unit costs. Costs associated with the maintenance of the intervention website and updating the website will also be included.

STIs prevented will be calculated by taking account of diagnoses recorded from clinical records at 12-month follow-up as well as self-reported episodes for the previous year. We will calculate the cost per episode of *Chlamydia* or *Gonorrhoea* prevented for the intervention group versus controls. The National Institute of Health and Care Excellence (NICE) recommends that quality-adjusted life years (QALYs) are used as the outcome in CEA, to allow for the comparison of results for different CEA across disease areas. QALYs are calculated by multiplying HRQoL by the amount of time spent in the HRQoL state. The EQ-5D is the questionnaire recommended by NICE to calculate HRQoL;^24^ it has been recognised, however, that the EQ-5D may not be suitable for economic evaluations of public health interventions as it may not capture the relevant information on the full psychosocial impact of public health interventions or be sufficiently sensitive for that purpose.^36^ ^37^ We will therefore also collect data on the performance of the Sexual QoL questionnaire^25^ to assess its suitability for use in a future large-scale RCT.

Acquisition of STI may have cost and QALYs impacts that occur beyond the end of the trial, so it is important that this information is accounted for as part of the model. This is commonly achieved by a decision analytical model that has a time horizon beyond the end of the trial and combines cost and outcome data from a range of published sources in addition to trial information. As a result, we will design a decision analytical model that will take account of costs and QALYs for the lifetime of the service users. The values in the decision analytical model will come from a comprehensive review of the literature including the efficacy of condoms, research to increase condom use and the incidence and prevalence of STIs. The quality of each type of evidence and relevance to the UK context will be assessed to determine the best coefficients to use in the cost-effectiveness model.^36^ We will also aim to determine utility values for the long-term QALYs outcomes associated with STIs. The final model will compare the incremental cost per QALYs gained and cost per STI prevented of the internet based intervention versus the control group. It will be subject to one-way, two-way and probabilistic sensitivity analyses and a cost-effectiveness acceptability curve calculated to determine the probability that the internet based intervention is cost-effective for a range of values of willingness to pay for an outcome gained.

## Ethical issues

### Potential ethical issues

This project aims to encourage behaviour change to reduce morbidity and the social and emotional costs of STI acquisition, with the aim of benefiting trial participants as well as wider society. There is a risk that the study may unintentionally exacerbate the stigma of STI and risky behaviour for participants. We strive to be non-judgemental about choices of lifestyle or behaviour, respecting others’ autonomy. It could be that participants’ partners or others see the intervention website, texts to participants’ mobile phones or email messages. Study information makes clear to participants the nature of study-related communications and possible risks. However, there is a danger that this may be accessed by others and that this leads to embarrassment or relationship difficulties in some way. A component of the intervention will focus on communication with partners, so it is hoped that the intervention will improve the quality of relationships rather than cause harm. Participants will receive detailed information about the study including risks and benefits while being led through the consent process on the trial software. Participants will be offered the opportunity to ask the researcher any further questions.

### Informed consent form and information sheet

Informed consent will be obtained using a standardised Participant Information Sheet and consent form (both integrated into the trial software), which have been approved by the London City and East ethics committee and local NHS Research and Development offices (see online supplementary appendix 1).

All participants included in the trial will be asked for their consent to take part and for their contact details to be used to communicate with them (eg, for follow-up questionnaires, and reminders to use the website), and for data obtained as a result of their use of the NHS services (ie, medical records) to be used for research purposes. Users will not be able to register for the study unless they consent to all statements. We will ensure that all research procedures meet the highest standards for data protection and confidentiality, storing data on an encrypted server. We will give participants the contact details for support organisations in case they are needed, and follow protocols to ensure the safety and well-being of participants under the age of 18 who may be at risk of harm.

### Protocol amendments

Protocol amendments will be communicated to the funder (NIHR Health Technology Assessment programme), the Trial Steering Committee, the Trial Management Group, the research sponsor and to the research ethics committee.

### Ancillary and post-trial care

Trial participants who request help will be advised to contact appropriate healthcare organisations (eg, their general practitioner) and offered the details of appropriate telephone helplines or online resources. All trial participants (including those allocated to the control group) will be offered access to the MenSS website at the end of the trial, for a period of 3 months.

## Dissemination

The following papers will be prepared and submitted for publication in peer-reviewed journals:

- Description of the development process of the MenSS intervention website;
- Description of the content of the MenSS intervention website;
- Main findings of the pilot trial;
- Findings of a qualitative process evaluation of trial procedures;
- Exploration of the relationship between mediators of condom use behaviour and outcome measures.

Findings will also be made available to participants and members of the public via Twitter and via a weblink from the University College London eHealth Unit website.
